# Supplementary material for: Clinical outcomes with lower versus conventional dose polymyxin B regimens in dialysis dependent and non-dialysis patients with gram-negative sepsis: A real-world propensity-score matched cohort study
Source: PLoS One. 2026 Mar 4;21(3):e0342835. doi: 10.1371/journal.pone.0342835 (PMC12959684; doi:10.1371/journal.pone.0342835)
Supplement: S1 Table — (DOCX) [file pone.0342835.s001.docx]

**S1_Table. Missing number for included variables**

| Variables | Missing, n (%), (n=674) |
| --- | --- |
| C-Reactive Protein (CRP) | 5 (0.007%) |
| Alkaline phosphatase (ALT) | 8 (0.011%) |
| Total bilirubin (TB) | 4 (0.005%) |
| Albumin (Alb) | 7 (0.01%) |
