# Supplementary material for: Clinical outcomes with lower versus conventional dose polymyxin B regimens in dialysis dependent and non-dialysis patients with gram-negative sepsis: A real-world propensity-score matched cohort study
Source: PLoS One. 2026 Mar 4;21(3):e0342835. doi: 10.1371/journal.pone.0342835 (PMC12959684; doi:10.1371/journal.pone.0342835)
Supplement: S2 Table — (DOCX) [file pone.0342835.s002.docx]

**S2_Table. Covariate balance after propensity score matching assessed using standardized mean differences (SMDs)**

| **Variables** | **Low dose Vs usual dose** | **Low dose Vs high dose** | **Usual dose Vs high dose** |
| --- | --- | --- | --- |
| APACHE II score | 0.024 | 0.017 | 0.071 |
| SOFA score | 0.075 | 0.046 | 0.014 |
| CCI | 0.005 | 0.079 | 0.010 |
| Dialysis requirement | 0.036 | 0.081 | 0.069 |

*APACHE II: Acute physiological and chronic health evaluation; SOFA: sequential organ failure assessment; CCI: Charlsons comorbidity index*
