# Supplementary material for: Clinical outcomes with lower versus conventional dose polymyxin B regimens in dialysis dependent and non-dialysis patients with gram-negative sepsis: A real-world propensity-score matched cohort study
Source: PLoS One. 2026 Mar 4;21(3):e0342835. doi: 10.1371/journal.pone.0342835 (PMC12959684; doi:10.1371/journal.pone.0342835)
Supplement: S3 Table — (DOCX) [file pone.0342835.s003.docx]

**S3_Table. Baseline study characteristics of the patients between low and usual group (post-matching)**

| **Variables** | **Low dose (n=179)** | **Usual dose (n= 179)** | ***p value*** |
| --- | --- | --- | --- |
| Age | 56 (19-89) | 58 (19-89) | 0.523 |
| Age category  18-30:  31-50:  51-70:  71-90:  >91: | 30 (16.76)  35 (19.55)  83 (48.72)  28 (15.64)  3(1.68) | 16 (8.9)  42 (23.4)  91 (50.8)  30 (16.75)  - | **0.002** |
| Gender  Male  Female | 102 (56.98)  77 (43.02) | 120 (67)  59 (32.9) | **0.0041** |
| Hypertension | 70 (39.11) | 82 (45.81) | 0.351 |
| Diabetes mellitus  Type 2 Diabetes Mellitus  T2DM + Diabetic Ketoacidosis | 63 (35.2)  - | 79 (44.13)  2 (1.11) | 0.324 |
| Cardiovascular Disease  Dyslipidemia  Heart failure  Ischemia Heart Disease  Rheumatoid Heart Disease | 1 (0.56)  4 (2.23)  14 (7.82)  1 (0.56) | 1 (0.5)  9 (5.02)  19 (10.61)  2 (1.11) | 0.412 |
| Cerebro Vascular Accident | 12 (6.7) | 9 (5.02) | 0.241 |
| Renal disease  Acute Kidney Injury  Chronic Kidney Disease | 1 (0.56)  21 (11.73) | 1 (0.5)  23 (12.8) | 0.412 |
| Liver disease  Chronic Liver Disease  Decompensated Chronic Liver Disease  DCLD with Pulmonary Hypertension  Hepatitis | 3 (1.68)  3 (1.68)  1 (0.56)  5 (2.79) | 3 (1.67)  -  3 (1.67)  4 (2.23) | 0.362 |
| Respiratory disease  Bronchial Asthma  Chronic Obstructive Pulmonry Disease  Interstitial Lung Disease | 5 (2.79)  13 (7.26)  - | 3 (1.67)  8 (4.46)  - | **0.0025** |
| Malignancy  Any metastatic carcinoma  Any solid tumor  Leukemia  Lymphoma  Myeloma | 1 (0.56)  11 (6.15)  3 (1.68)  2 (1.12)  1 (0.56) | 2 (1.11)  9 (5.02)  4 (2.23)  2 (1.11)  - | **0.004** |
| Hypothyroidism | 14 (7.82) | 12 (6.7) | 0.354 |
| History of transplantation | 1 (0.56) | 2 (1.11) | 0.418 |
| Past surgical history | 46 (25.7) | 49 (27.27) | 0.652 |
| Pallor | 63 (35.2) | 46 (25.69) | **0.003** |
| Icterus | 19 (10.61) | 13 (7.26) | 0.325 |
| Cyanosis | 1 (0.56) | 1 (0.55) | 0.842 |
| Clubbing | 2 (1.12) | 2 (1.11) | 0.362 |
| Lymphadenopathy | 3 (1.68) | 1 (0.55) | 0.145 |
| Edema | 38 (21.23) | 34 (18.99) | 0.862 |
| Glascow Coma Scale | 15 (3-15) | 15 (3-15) | **0.001** |
| Transferred from outside hospital | 52 (29.05) | 60 (33.51) | 0.362 |
| History of recent admission | 20 (11.17) | 23 (12.84) | 0.412 |
| Resistance pattern  Susceptible  Multi Drug Resistance  Extended Drug Resistance | 31 (17.32)  13 (7.26)  128 (71.51) | 28 (15.64)  9 (5.02)  133 (74.3) | 0.325 |
| Pathogens  CRAB  CRE  CRPA | 52 (29.37)  28 (15.81)  25 (14.12) | 81 (45.25)  49 (27.37)  46 (25.69) | 0.146 |
| Combination therapy with polymyxin B  Tigecycline  Cefta-Avi+Aztreonam  Meropenem  Other β lactams | 14 (7.9)  12 (6.7)  8 (4.5)  16 (9.03) | 16 (8.93)  11 (6.14)  8 (4.46)  10 (5.58) | 0.325 |

*APACHE II: Acute physiological and chronic health evaluation; SOFA: sequential organ failure assessment; CRAB: carbapenem resistant Acinetobacter baumannii; CRE: carbapenem resistant Enterobacteriaceae; CRPA: carbapenem resistant pseudomonas aeruginosa*
