# Supplementary material for: Clinical outcomes with lower versus conventional dose polymyxin B regimens in dialysis dependent and non-dialysis patients with gram-negative sepsis: A real-world propensity-score matched cohort study
Source: PLoS One. 2026 Mar 4;21(3):e0342835. doi: 10.1371/journal.pone.0342835 (PMC12959684; doi:10.1371/journal.pone.0342835)
Supplement: S4 Table — (DOCX) [file pone.0342835.s004.docx]

**S4_Table. End point outcomes after polymyxin B therapy in the dialysis requiring cohort patients (after propensity score matching, n=254)**

| **Clinical outcomes** | **Usual Vs Low dose (n= 130)** | | **High Vs Low dose (n= 62)** | | **High Vs Usual dose (n= 62)** | |
| --- | --- | --- | --- | --- | --- | --- |
|  |  | *p value* |  | *p value* |  | *p value* |
| 28-day mortality [Cox proportional hazard (95% CI)] | 1.21 (0.79-1.85) | 0.382 | 1.23 (0.63-2.40) | 0.314 | 1.22 (0.68-2.20) | 0.944 |
| Microbiological clearance [Odds ratio (95% CI)] | **1.27 (1.06-2.21)** | **0.006** | 1.24 (0.50-3.12) | 0.646 | 1 (0.42-2.41) | 1.00 |
| Ventilator free days (Median IQR) | 8.6 (0-106) vs 2 (0-76) | **0.002** | 8.6 (0-106) vs 4.5 (0-56) | 0.37 | 2 (0-76) vs 4.5 (0-56) | 0.46 |
| ICU free days (Median IQR) | 9.8 (0-111) vs 3.5 (0-77) | **0.047** | 9.8 (0-111) vs 5 (0-56) | 0.48 | 3.5 (0-77) vs 5 (0-56) | 0.58 |
| Vasopressor free days (Median IQR) | 3 (0-46) vs 3 (0-50) | **0.05** | 3 (0-46) vs 4 (0-24) | 0.69 | 3 (0-50) vs 4 (0-24) | 0.86 |
