# Supplementary material for: Clinical outcomes with lower versus conventional dose polymyxin B regimens in dialysis dependent and non-dialysis patients with gram-negative sepsis: A real-world propensity-score matched cohort study
Source: PLoS One. 2026 Mar 4;21(3):e0342835. doi: 10.1371/journal.pone.0342835 (PMC12959684; doi:10.1371/journal.pone.0342835)
Supplement: S6 Table — (DOCX) [file pone.0342835.s006.docx]

**S6_Table. Sensitivity analysis for 28-day mortality for all the included cohort patients after polymyxin B therapy (after propensity score matching using pairwise 1:3 nearest neighbour matching)**

| **Clinical outcomes** | **Usual Vs Low dose (n= 349)** | | **High Vs Low dose (n= 221)** | | **High Vs Usual dose (n= 297)** | |
| --- | --- | --- | --- | --- | --- | --- |
|  |  | *p value* |  | *p value* |  | *p value* |
| 28-day mortality [Cox proportional hazard (95% CI)] | **1.48 (1.12-1.96)** | **0.006** | 1.22 (0.84-1.78) | 0.29 | 1.15 (0.82-1.61) | 0.415 |
