# Supplementary material for: Clinical outcomes with lower versus conventional dose polymyxin B regimens in dialysis dependent and non-dialysis patients with gram-negative sepsis: A real-world propensity-score matched cohort study
Source: PLoS One. 2026 Mar 4;21(3):e0342835. doi: 10.1371/journal.pone.0342835 (PMC12959684; doi:10.1371/journal.pone.0342835)
Supplement: S7 Table — (DOCX) [file pone.0342835.s007.docx]

**S7_Table. Subgroup analysis for 28-day mortality for all the included cohort patients after polymyxin B therapy categorized based on gender (after propensity score matching)**

| **Clinical outcomes** | **Usual Vs Low dose (n= 144)** | | **High Vs Low dose (n= 44)** | | **High Vs Usual dose (n= 44)** | |
| --- | --- | --- | --- | --- | --- | --- |
|  |  | *p value* |  | *p value* |  | *p value* |
| Male [28-day mortality {Cox proportional hazard (95% CI)}] | **1.56 (1.076-2.267)** | **0.018** | 1.392 (0.762-2.545) | 0.282 | 1.236 (0.749-2.039) | 0.406 |
| Female [28-day mortality {Cox proportional hazard (95% CI)}] | **1.585 (1.013-2.479)** | **0.044** | 1.06 (0.465-2.41) | 0.89 | 0.55 (0.249-1.22) | 0.14 |
