# Supplementary material for: Clinical outcomes with lower versus conventional dose polymyxin B regimens in dialysis dependent and non-dialysis patients with gram-negative sepsis: A real-world propensity-score matched cohort study
Source: PLoS One. 2026 Mar 4;21(3):e0342835. doi: 10.1371/journal.pone.0342835 (PMC12959684; doi:10.1371/journal.pone.0342835)

**S1_Fig. Culture reports pre- and post-polymyxin B therapy with various dosing strategies among all the included cohort patients (i) Blood (ii) endotracheal tube (iii) urine (iv) wound swab (v) broncho-alveolar lavage (vi) Tissue (vii) body fluids (viii) catheter tip (ix) pus (x) sputum (xi) nasal swab**


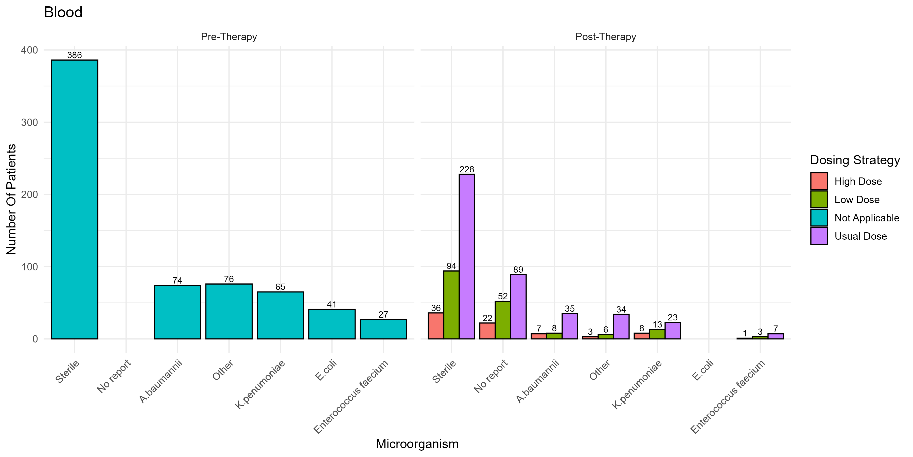


*
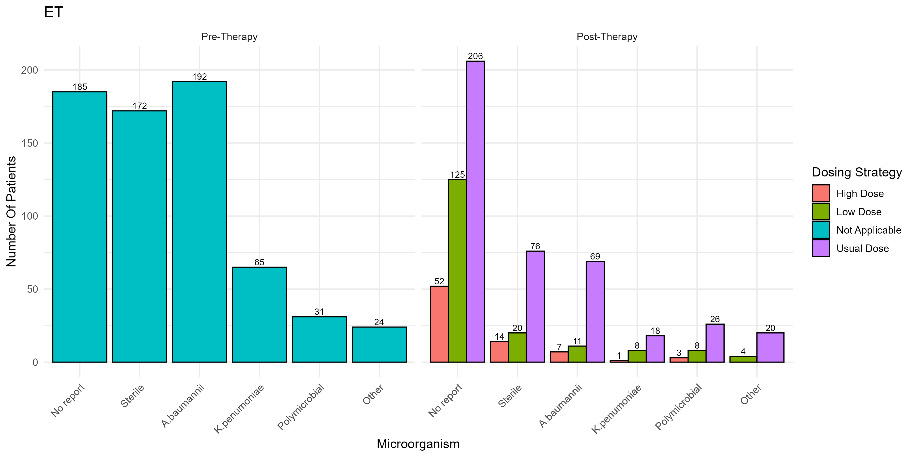
*


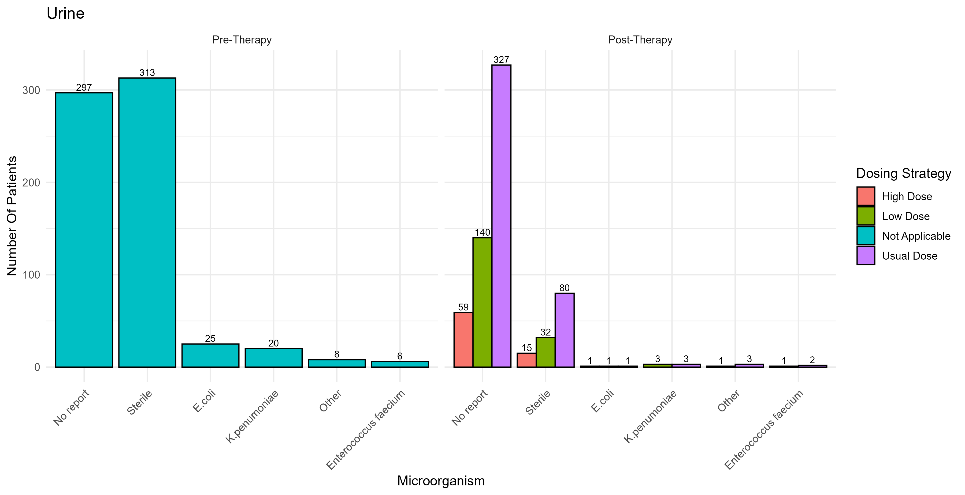


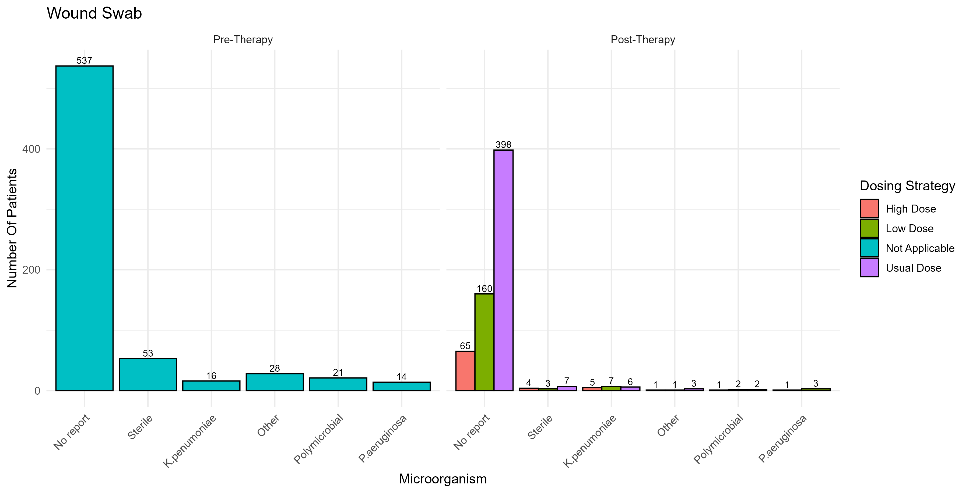


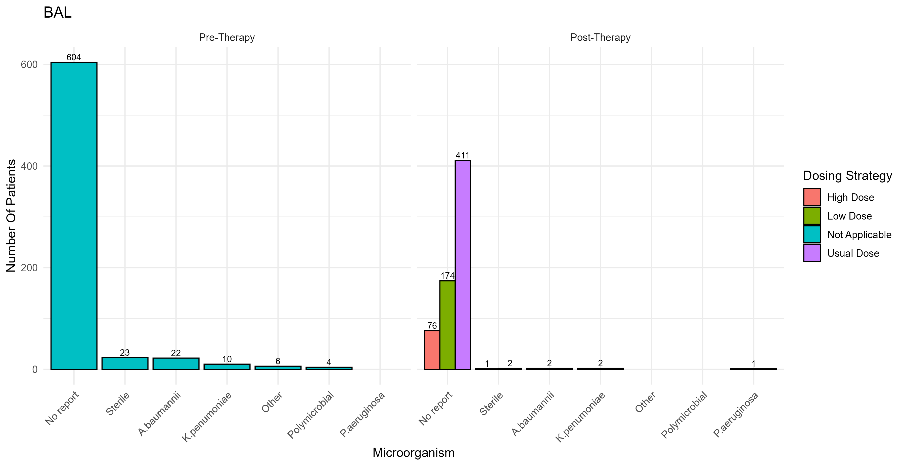


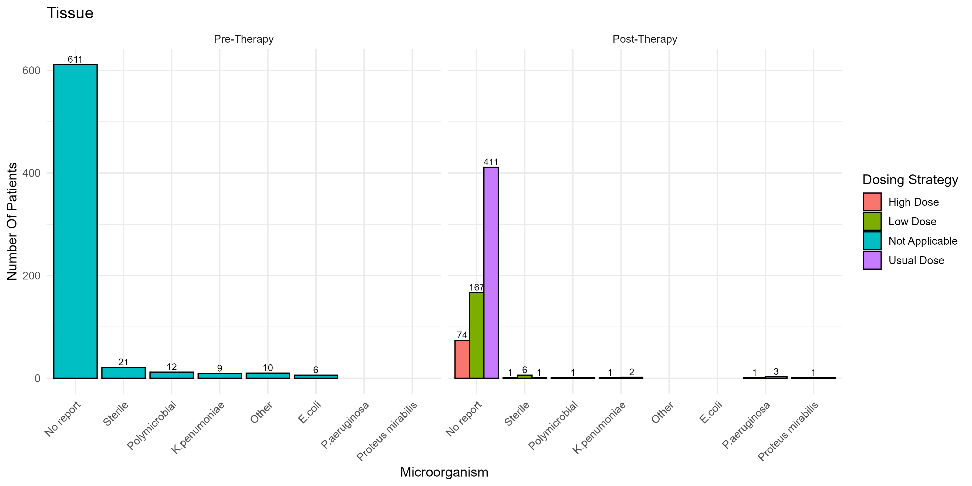


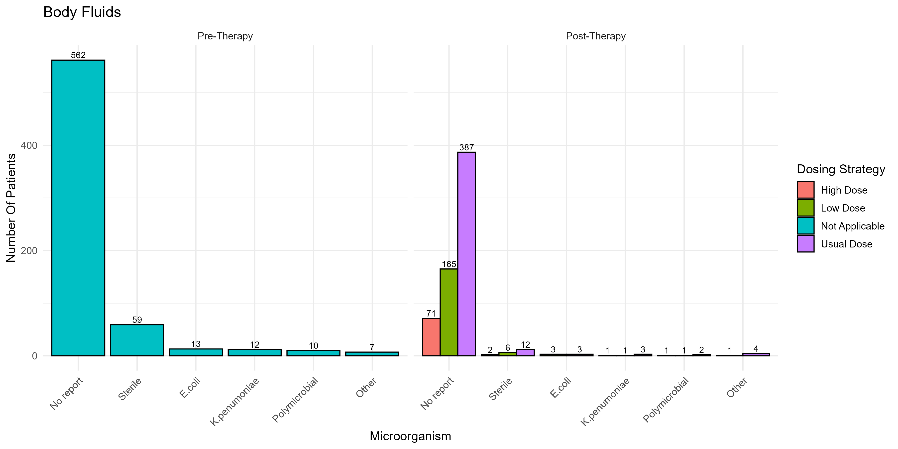


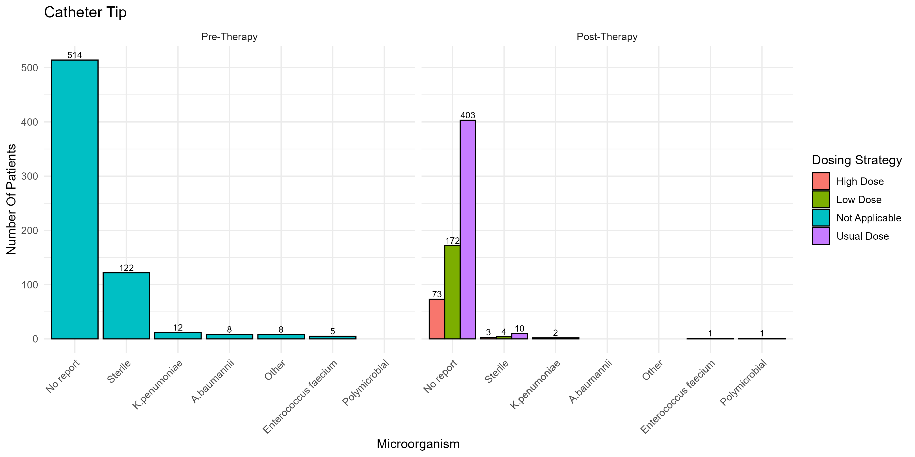


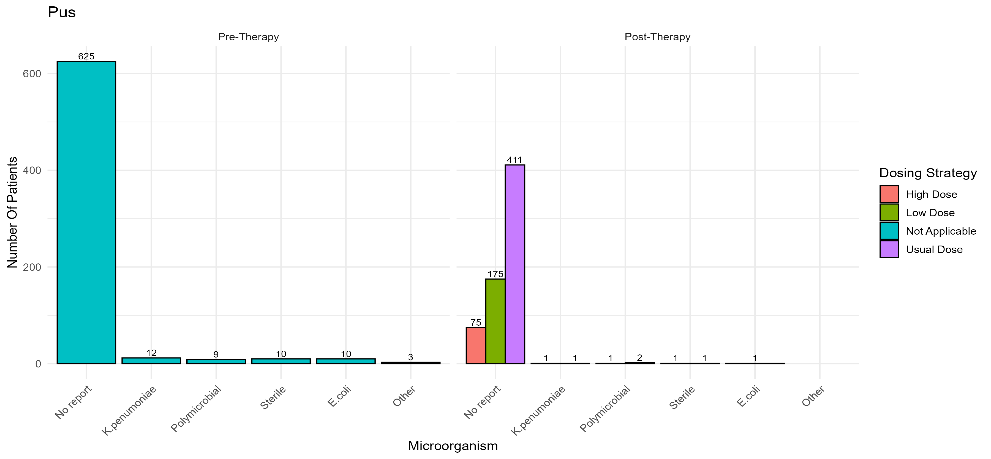


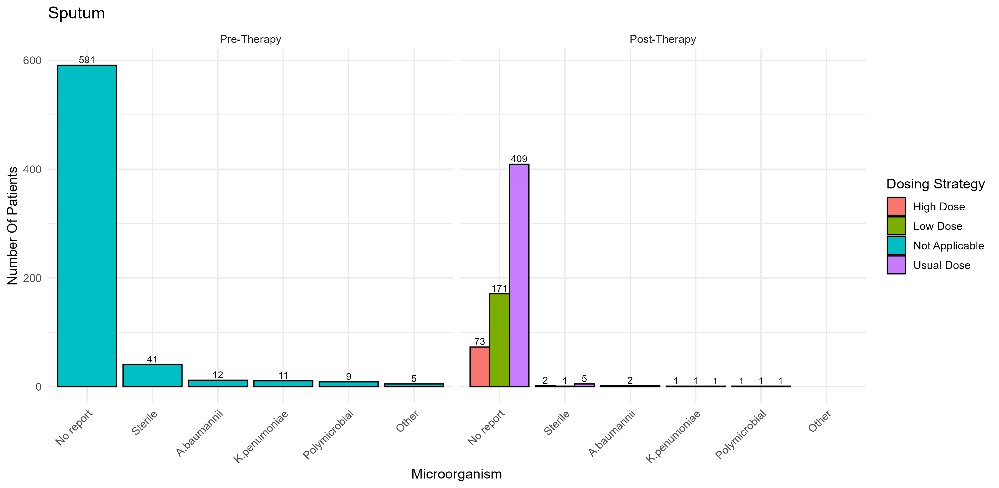


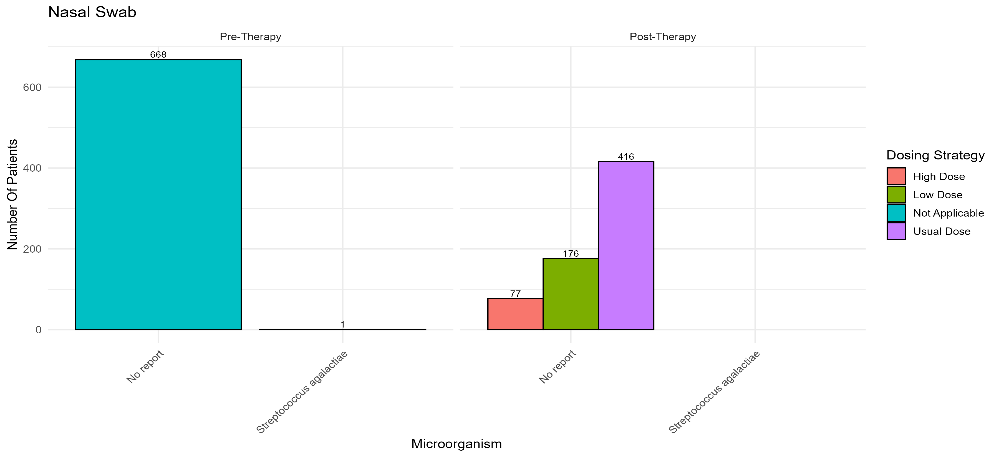

Supplement: S1 Fig — (DOCX) [file pone.0342835.s008.docx]
