# Supplementary material for: Clinical outcomes with lower versus conventional dose polymyxin B regimens in dialysis dependent and non-dialysis patients with gram-negative sepsis: A real-world propensity-score matched cohort study
Source: PLoS One. 2026 Mar 4;21(3):e0342835. doi: 10.1371/journal.pone.0342835 (PMC12959684; doi:10.1371/journal.pone.0342835)

**S2_Fig. Kaplan Meier analysis of 28-mortality status, 28-day mortality between patients requiring dialysis comparing low, usual and high dosing strategy of polymyxin B after matching. (A) Low dose Vs. Usual dose (B) Low dose Vs High dose (C) Usual dose Vs High dose**


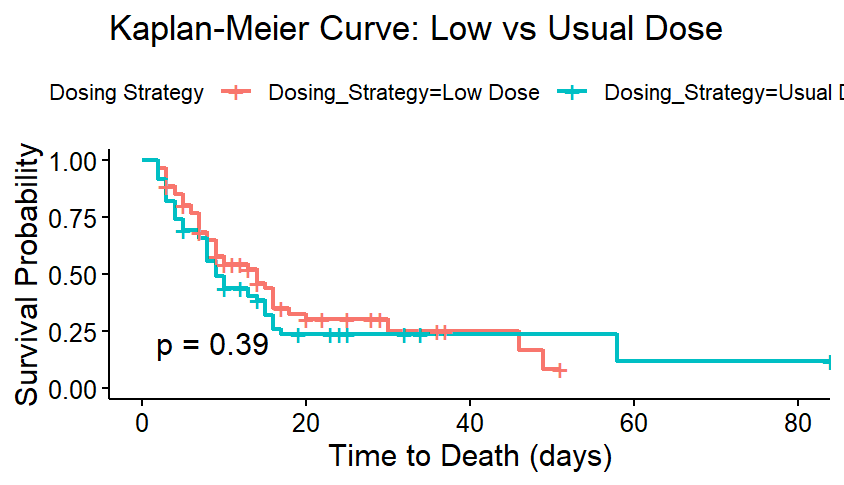


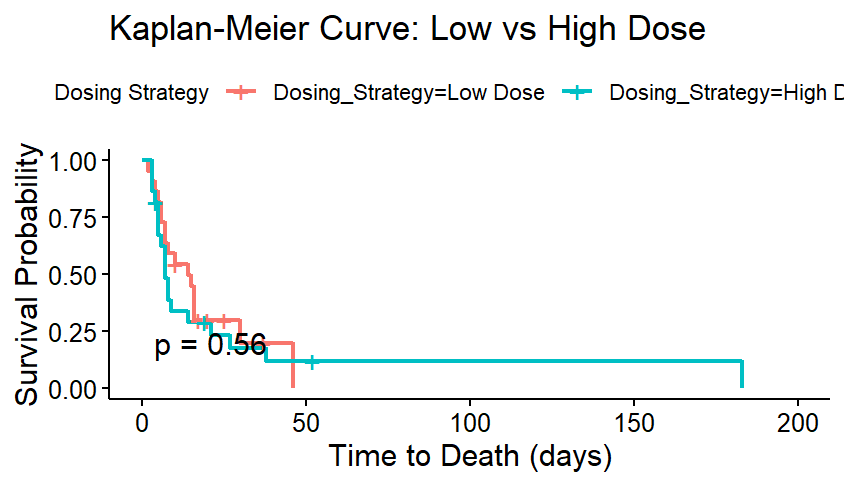


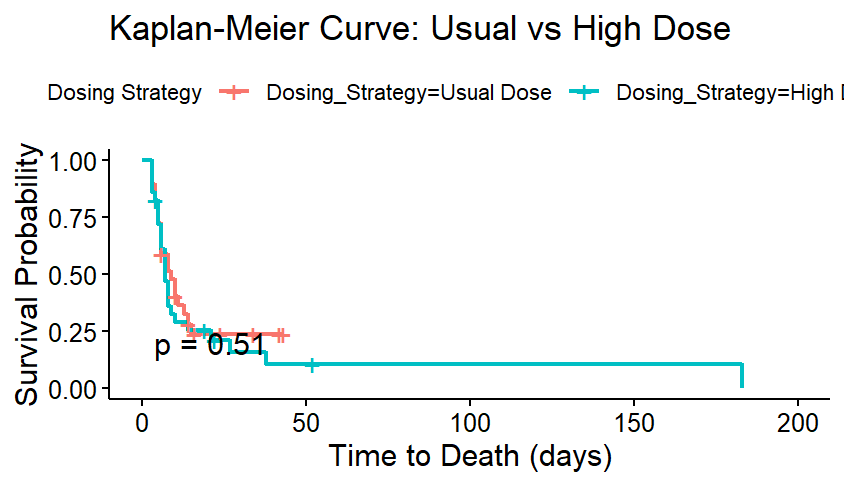

Supplement: S2 Fig — (A) Low dose Vs Usual dose (B) Low dose Vs High dose (C) Usual dose Vs High dose. (DOCX) [file pone.0342835.s009.docx]
