# Supplementary material for: Clinical outcomes with lower versus conventional dose polymyxin B regimens in dialysis dependent and non-dialysis patients with gram-negative sepsis: A real-world propensity-score matched cohort study
Source: PLoS One. 2026 Mar 4;21(3):e0342835. doi: 10.1371/journal.pone.0342835 (PMC12959684; doi:10.1371/journal.pone.0342835)
Supplement: S3 Fig — (A) Hemodialysis (B) Sustained low efficiency dialysis (C) Hemodialysis + Sustained low efficiency dialysis. (DOCX) [file pone.0342835.s010.docx]

**S3_Fig. Kaplan Meier analysis of 28-mortality status, 28-day mortality between patients with various dosing strategies requiring different types of dialysis, comparing low, usual and high dosing strategy of polymyxin B after matching. (A) Hemodialysis (B) Sustained low efficiency dialysis (C) Hemodialysis + Sustained low efficiency dialysis**


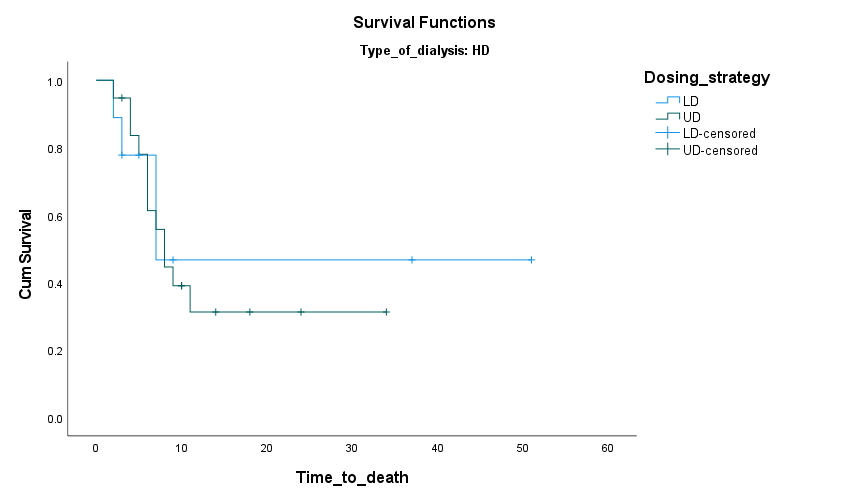
p=0.771


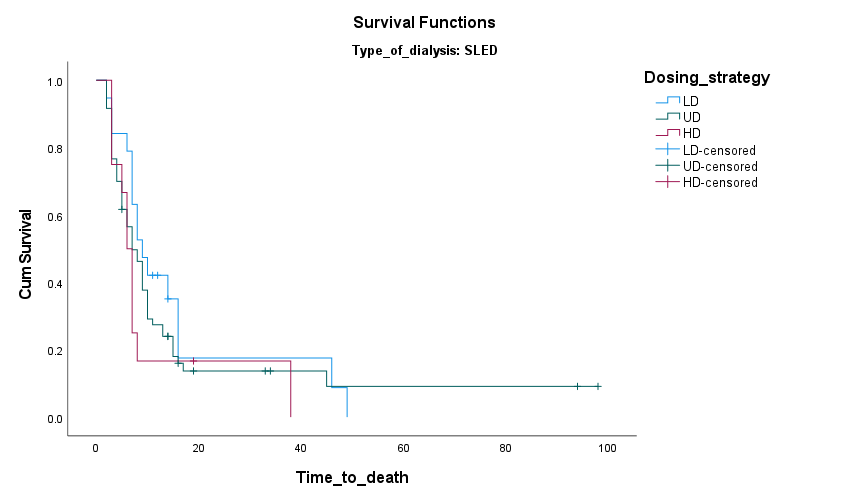
p=0.417


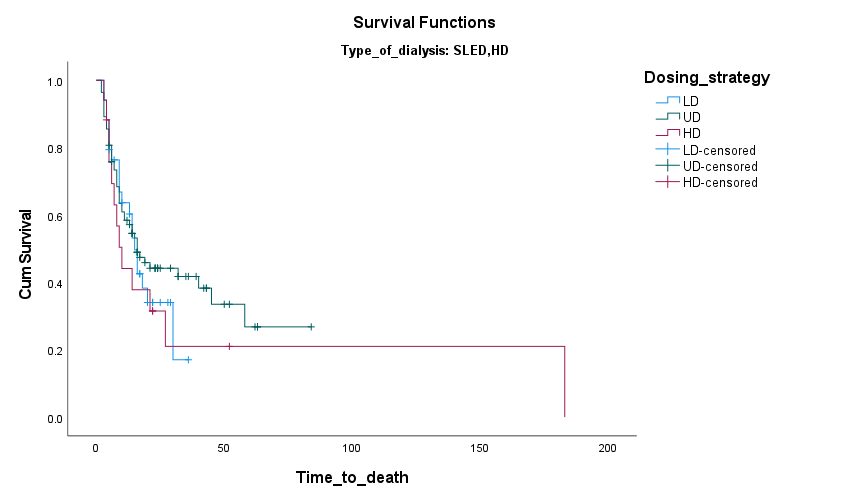
p=0.539
