# Supplementary material for: Clinical outcomes with lower versus conventional dose polymyxin B regimens in dialysis dependent and non-dialysis patients with gram-negative sepsis: A real-world propensity-score matched cohort study
Source: PLoS One. 2026 Mar 4;21(3):e0342835. doi: 10.1371/journal.pone.0342835 (PMC12959684; doi:10.1371/journal.pone.0342835)
Supplement: Attachment — Submitted filename: Comments for Manuscript (04.09.2025).docx [file pone.0342835.s011.docx]

**MAJOR REVISIONS**

**a) Study Design and Methodology**

- **Retrospective nature:** The authors acknowledge this, but more detail is needed about how missing or inconsistent clinical data were handled (e.g., infection source documentation, microbiological clearance definitions).
- **Exposure categorization:** The rationale for defining "low," "usual," and "high" dosing cut-offs (Table I) should be better justified with reference to guideline standards or prior clinical literature. At present, these thresholds appear somewhat arbitrary.
- **Dialysis subgroup:** Although subgroup analyses are presented, the heterogeneity of dialysis modalities (SLED, CRRT, HD) is high. The manuscript should provide more clarity on how these modalities were distributed across groups and whether these confounded outcomes.
- **Confounders:** Propensity score matching was performed, but key covariates (e.g., pathogen MIC values, concurrent nephrotoxic drugs) were not included. The authors should discuss how unmeasured confounding might influence results.

**b) Statistical Analysis**

- The manuscript uses Cox regression, Kaplan–Meier survival, and logistic regression appropriately. However:
  - Confidence intervals should be reported consistently for all effect sizes (not only HRs).
  - The choice of caliper width for propensity score matching (0.1 SD) should be justified.
  - Post-hoc power analysis adds little value; more emphasis should be placed on observed effect sizes and their precision.

**c) Results Interpretation**

- The finding that usual dosing was associated with higher mortality than low dosing is counterintuitive. The authors should explore potential explanations (e.g., confounding by severity, higher toxicity, clinician bias in assigning higher doses to sicker patients).
- Microbiological clearance data are limited; since clearance rates were low across groups, the conclusion of "non-inferiority" seems overstated. The authors should reframe this as "no significant difference observed" rather than formal non-inferiority.

**d) Discussion and Conclusions**

- The discussion is generally balanced but occasionally overstates the strength of evidence. Phrases like “supports individualized, lower dosing strategies” should be qualified as hypothesis-generating pending prospective confirmation.
- Comparison with other recent clinical and pharmacokinetic studies (e.g., therapeutic drug monitoring-guided trials, 2022–2024) could be expanded.

**MINOR REVISIONS**

**Abstract:** The results section should report actual mortality percentages in each group for clarity.

**Figures/Tables:** Figures lack detailed legends, making them difficult to interpret independently.

**Terminology:** Ensure consistency in abbreviations (e.g., “LU” vs. “lakh units”; sometimes spelled inconsistently).

**References:** The reference list is up-to-date, but some statements in the Discussion (e.g., inter-individual variability in Polymyxin B pharmacokinetics) could benefit from citing very recent studies (2023–2025).
