# Supplementary material for: Clinical outcomes with lower versus conventional dose polymyxin B regimens in dialysis dependent and non-dialysis patients with gram-negative sepsis: A real-world propensity-score matched cohort study
Source: PLoS One. 2026 Mar 4;21(3):e0342835. doi: 10.1371/journal.pone.0342835 (PMC12959684; doi:10.1371/journal.pone.0342835)
Supplement: Attachment — Submitted filename: Response to Reviewers.docx [file pone.0342835.s013.docx]

**Response to reviewers**

**Reviewer #1:**

This is a well-conducted, large, real-world retrospective cohort study addressing a significant clinical dilemma: the optimal dosing of polymyxin B in critically ill patients, particularly those on dialysis. The methodology is robust, employing propensity score matching to mitigate confounding, and the results are provocative, suggesting lower doses may be non-inferior or even superior to guideline-recommended doses. The manuscript is generally well-written and structured. However, several major limitations, inherent to its retrospective design, necessitate careful consideration and temper the strength of the conclusions. The study evaluates both primary (28-day mortality) and important secondary outcomes (microbiological clearance, ventilator-/vasopressor-/ICU-free days), providing a comprehensive view of efficacy and clinical utility. The separate analysis of the 254 dialysis-dependent patients is a key strength, as this is the population where dosing is most controversial and pharmacokinetics are most complex.

***Response:***

We sincerely thank the reviewer for their thorough and insightful evaluation of our work. We appreciate the recognition of the study’s strengths. We have modified the manuscript accordingly addressing each comment.

***Comment 1:***

The Fundamental Issue of Confounding by Indication (Unmeasured Confounding): This is the most significant threat to the validity of the conclusions. While propensity score matching adjusted for measured confounders like severity scores (APACHE II, SOFA), it cannot account for unmeasured ones. Crucially, the "dosing strategy" was at the physician's discretion. It is highly plausible that clinicians intentionally used lower doses in patients they perceived as more fragile, with poorer prognoses, or at higher risk of toxicity (e.g., more multi-organ dysfunction not fully captured by SOFA). Conversely, they may have used higher doses in patients they were "fighting for" or in those with perceived more severe infections. This would bias the results against the low-dose group (making them look worse) and in favor of the high-dose group (making them look better). The fact that the low-dose group had better outcomes despite this potential bias is striking, but it does not eliminate the concern. This inherent limitation of observational studies can only be definitively resolved by an RCT.

***Response:***

We fully agree with the reviewer that confounding by indication is a major concern in retrospective observational studies and that propensity score matching cannot eliminate unmeasured confounders. It is highly likely that physician’s discretion influenced dosing strategies (e.g., prescribing lower doses for more fragile patients or higher doses for those with more severe infection). We have explicitly acknowledged this in the revised Discussion and Limitations. Importantly, we note that despite this potential bias against the low-dose group, outcomes were not inferior in this group, which makes the finding noteworthy but also underscores the need for prospective randomized trials to definitively answer this question.

*“Discussion: Confounding by indication is the most plausible explanation, as clinicians may have preferentially prescribed higher doses to patients perceived as more severely ill or with poor prognostic indicators not fully captured by APACHE II or SOFA scores. Additionally, the potential for dose-related adverse effects, particularly nephrotoxicity in patients with renal impairment, cannot be excluded. Local fixed dose prescribing practices may also have resulted in inadvertent overdosing in smaller patients, amplifying toxicity risk. Collectively, these factors could explain the paradoxical association and underscore the need for randomized controlled trials to delineate true dose-outcome relationships.*

*Limitation: Although propensity score matching was performed to minimize bias from measured variables, residual and unmeasured confounding cannot be excluded. Physician discretion in dose selection, based on clinical impressions not captured by APACHE II or SOFA scores, may have influenced outcomes.”*

***Comment 2:***

Definition of Dosing Groups: The definitions in Table I are unconventional and potentially problematic. "Usual dose" is defined as a fixed dose (150mg/15LU Q12H), not a weight-based one. Current guidelines recommend 1.25-1.5 mg/kg Q12H. For an 80kg patient, this would be 100-120mg Q12H. The study's "usual dose" (150mg Q12H) is actually a high weight-based dose for most patients. This misclassification likely contaminates the groups and makes interpretation challenging. The "high dose" group might represent extreme outliers or dosing errors. The grouping is based on both loading and maintenance dose. It is unclear how patients were categorized if, for example, they received a high loading dose but a low maintenance dose. A more granular analysis of total cumulative dose or average daily dose might be more informative.

***Response:***

We thank the reviewer for this important observation. We agree that our definition of dosing groups was unconventional, as it was based on fixed-dose regimens commonly used in our centre rather than guideline-recommended weight-based dosing. This reflects the reality of prescribing practice during the study period, where doses were routinely documented in lakh units (LU) and patient-level body weight was often not recorded. Because of this limitation, we were unable to perform a formal mg/kg-based analysis. We have clarified this in the revised Methods and explicitly acknowledged the potential for misclassification bias in the Limitations. Patients who received different loading and maintenance regimens were categorised based on their maintenance dose, as this reflects ongoing exposure.

“*Methods: Dosing categories were defined according to the prescribing practices at our centre, where doses are routinely documented in lakh units (LU) rather than weight-based regimens. For categorization, we adopted thresholds reflecting the most frequently observed patterns in clinical practice. We categorized polymyxin B dosing into three strategies- “low”, “usual” and “high”- with specific loading and maintenance regimens as defined in Table I. Although these definitions differ from international guideline recommendations, [weight-based dosing (LD 2.0-2.5 mg/Kg; MD1.25-1.5mg/kg q12h)], they are consistent with prior observational studies that have reported fixed-dose regimens in similar settings*[1]*. Unfortunately, patient-level body weight was not consistently recorded in our dataset, preventing a formal mg/kg-based analysis. Patients were classified according to the predominant dosing regimen used during therapy. In cases where loading and maintenance doses differed (eg. High loading dose but usual maintenance dose), categorization was based on the maintenance regimen, as this reflects ongoing exposure. We used fixed-dose categories because patient-level body weight data were not consistently available in the medical records. Moreover, it was often impractical to obtain accurate weight measurements for patients admitted to the ICU in critical condition due to the unavailability of high-end patient beds in the ICU, thereby precluding dose recalculation in mg/kg as recommended by clinical guidelines; precluding recalculation of doses in mg/Kg as recommended by guidelines. This approach therefore reflects actual prescribing and documentation practices in our setting rather than an attempt to replace weight-based pharmacological principles.*

*Limitation: Body weight data were largely unavailable, preventing normalization of dose on a mg/kg basis and potentially introducing misclassification bias when categorizing fixed-dose regimens.”*

***Comment 3:***

Lack of Toxicity Data: For a study arguing for lower dosing, the omission of toxicity outcomes (especially nephrotoxicity and neurotoxicity) is a major weakness. The primary rationale for lower dosing is to reduce harm. Demonstrating non-inferior efficacy with significantly reduced toxicity would be a much more powerful and clinically relevant conclusion. The authors had the data to analyze this (e.g., rates of new AKI, need for dialysis) but did not report it.

***Response:***

We thank the reviewer for highlighting this important point. We fully agree that the evaluation of toxicity is critical when comparing different polymyxin B dosing strategies, particularly since lower dosing is often advocated to reduce nephrotoxicity and neurotoxicity. We have now clarified this explicitly in the Methods, discussion, limitations and future implications sections.

*“Methods: All available data on the safety and effectiveness parameters of polymyxin B were retrieved from patients medical records and systematically analyzed.*

*Discussion:* *We observed all cases of acute kidney injury (AKI) reported in the medical records following the initiation of polymyxin B therapy. However, due to the retrospective design of the study, it was not possible to determine whether the reported AKI was attributable to the disease itself or to the drug. Furthermore, a causal relationship between AKI and polymyxin B administration could not be established, as no dechallenge or rechallenge was performed. This limitation is particularly important given the critical condition of the study population, in which multiple concurrent factors could have contributed to renal dysfunction.*

*Limitations: Furthermore, pharmacokinetic data to correlate administered doses with serum drug concentrations or toxicity endpoints were lacking, and systematic assessment of nephrotoxicity and neurotoxicity could not be performed. The absence of these data precluded evaluation of exposure-response relationships and dose-related toxicity.*

*Future implications: Also, they could incorporate standardized toxicity endpoints to better delineate the benefit-risk profile of different dosing strategies.”*

***Comment 4:***

Microbiological Data Limitations: The lack of MIC data is a notable limitation. It is impossible to know if the groups were balanced in terms of the severity of resistance (e.g., high MIC "creep" in one group). The microbiological clearance rate is also surprisingly low across all groups (~12-15%), raising questions about the definitions used or the inherent difficulty of clearing these infections.

***Response:***

We thank the reviewer for these valuable observations. We acknowledge that the absence of MIC data is an important limitation and also the low microbiological clearance has been addressed in the methods, results and limitations section of the manuscript.

*“Methods: Microbiological clearance was defined as the absence of the baseline pathogen in follow-up cultures obtained ≥72 hours after starting polymyxin B therapy. with no subsequent isolation of the same organism* [2]*. Patients without repeat cultures were conservatively classified as “not cleared”. This stringent definition was chosen to minimize misclassification, but it may underestimate clearance compared with more permissive criteria. Minimum inhibitory concentrations were not uniformly available because susceptibility testing during the study period was routinely performed using disc diffusion methods, and MIC determination was only conducted selectively. Consequently, we were unable to assess whether treatment groups differed in the distribution of MIC values or to evaluate exposure-response relationships*

*Results: Microbiological clearance was very low across all dosing groups (12-15%), which likely reflects the severity of the infections and the inherent difficulty of achieving eradication in critically ill patients. In many cases, polymyxin B was initiated as salvage therapy at an advanced stage of sepsis, by which time microbiological clearance was rarely observed.*

*Limitations: Microbiological data such as infection source, MIC values and timing of follow-up cultures were not uniformly documented, which may have introduced classification bias when assessing clearance.”*

***Comment 5:***

Single-Center Study: Practices at this single tertiary-care center (e.g., specific dialysis protocols, common pathogens, prevailing prescribing habits) may not be generalizable to other institutions globally.

***Response:***

We thank the reviewer for pointing this out. We agree that our study reflects the practices of a single tertiary-care centre and that factors such as local prescribing habits, pathogen prevalence, resistance profiles, and dialysis protocols may limit generalizability. We have now explicitly acknowledged this in both the Discussion and Limitations.

*“Discussion: As a single-centre study, our results reflect local prescribing habits, patient mix and dialysis practices, which may limit generalizability. Nevertheless, they offer valuable real-world insight into polymyxin B use critically ill patients with renal impairment in a high burden setting and the urgent need for multicentre randomized trials to establish optimal, safe and effectives dosing strategies.*

*Limitation: This was a retrospective single-centre study, which may limit the generalizability of our findings, as local prescribing practices, pathogen distribution, and dialysis protocols may vary across institutions . However, the methodological approach employed to evaluate dosing strategies can be adapted and applied in future studies conducted in different settings.”*

***Comment 6:***

The results suggest that in this specific real-world setting, a strategy of using lower, fixed doses of polymyxin B was not associated with worse outcomes than using higher, guideline-derived doses. This is an important finding that challenges dogma and may reflect that the toxicity of higher doses offsets their potential efficacy benefits. However, the study cannot prove that low-dose therapy is non-inferior or superior due to the unmeasurable confounding by indication. The observed survival benefit might be because physicians correctly identified patients who would do well regardless of antibiotic dose and thus used a less aggressive, safer regimen.

***Response:***

We thank the reviewer for this thoughtful and constructive feedback. We fully agree that our study cannot establish non-inferiority or superiority of low dose polymyxin B due to the inherent problem of confounding by indication. We have therefore revised the Discussion to acknowledge this limitation and to frame our results as hypothesis-generating rather than definitive. We also clarified that physicians’ clinical judgement may have influenced dose selection, which could explain the observed survival patterns. Also, we have modified the title and conclusion section of the manuscript accordingly.

*“Discussion: Our results add a clinical perspective to these observations, emphasizing the importance to individualized dosing, particularly in patients with renal impairment or multi-organ dysfunction who are prone to drug accumulation. Although lower doses were not associated with worse outcomes in our cohort, the absence of systematic toxicity data precludes conclusions about safety benefits. Thus, the rationale for exploring lower-dose regimens remains hypothesis-generating and warrants confirmation in prospective studies that assess both efficacy and safety outcomes.*

*Conclusion: In this single-centre retrospective study, lower fixed doses of polymyxin B were associated with decreased mortality and better clinical outcomes when compared with higher doses. They may be feasible in critically ill patients with renal impairment, with limited safety data. However, given the inherent limitations of observational research, including unmeasured confounding and absence of systematic toxicity assessment, these results should be interpreted as hypothesis-generating rather than practice-changing. Our findings support the feasibility and importance of investigating lower dose polymyxin B regimens in future RCTs.*

*Title: Clinical outcomes with* *lower versus conventional dose polymyxin B regimens in dialysis dependent and non-dialysis patients with gram-negative sepsis: A real-world propensity-score matched cohort study”*

**Reviewer #2:**

This manuscript presents clinically relevant investigation into the optimal dosing of polymyxin B in a critically ill population, including a significant subset on dialysis. The study addresses an important knowledge gap with a robust sample size and sophisticated statistical methodology. However, several major concerns must be addressed before the manuscript can be considered for publication.

***Response:***

We sincerely thank the reviewer for their thoughtful and constructive feedback. We have now addressed each comment.

***Comment 1:***

The dosing categories are problematic and threaten the validity of the conclusions. The groups are defined by fixed doses (e.g., ≤150mg = "low") rather than weight-based dosing (mg/kg), which is the standard recommended in guidelines. A 150 mg dose is a "low" dose for a 100 kg patient but a "high" dose for a 50 kg patient.

The term "usual dose" is defined as exactly 150mg (15 LU) LD and MD. However, international guidelines recommend a weight-based loading dose of 2.0-2.5 mg/kg and a maintenance dose of 1.25-1.5 mg/kg every 12 hours. The study's definition is not "usual" according to the literature. So, the authors must re-analyze their data using weight-based dosing categories. Define groups based on mg/kg of actual body weight. This is essential for the results to be interpretable and generalizable.

***Response:***

We thank the reviewer for this important observation. We agree that guideline-recommended dosing is expressed in mg/kg, and that fixed-dose categories may not reflect the same exposure across different body weights. Unfortunately, patient-level weight data were not consistently available in our dataset, which precluded re-analysis based on mg/kg dosing. This limitation reflects the real-world prescribing and documentation practices at our centre, where polymyxin B is dispensed in lakh units (LU) and doses are typically prescribed in fixed amounts. We have clarified this in the revised Methods and explicitly acknowledged it in the Limitations.

“*Methods: Dosing categories were defined according to the prescribing practices at our centre, where doses are routinely documented in lakh units (LU) rather than weight-based regimens. For categorization, we adopted thresholds reflecting the most frequently observed patterns in clinical practice. We categorized polymyxin B dosing into three strategies- “low”, “usual” and “high”- with specific loading and maintenance regimens as defined in Table I. Although these definitions differ from international guideline recommendations, [weight-based dosing (LD 2.0-2.5 mg/Kg; MD1.25-1.5mg/kg q12h)], they are consistent with prior observational studies that have reported fixed-dose regimens in similar settings*[1]*. Unfortunately, patient-level body weight was not consistently recorded in our dataset, preventing a formal mg/kg-based analysis. Patients were classified according to the predominant dosing regimen used during therapy. In cases where loading and maintenance doses differed (eg. High loading dose but usual maintenance dose), categorization was based on the maintenance regimen, as this reflects ongoing exposure. We used fixed-dose categories because patient-level body weight data were not consistently available in the medical records. Moreover, it was often impractical to obtain accurate weight measurements for patients admitted to the ICU in critical condition due to the unavailability of high end patient beds in the ICU, thereby precluding dose recalculation in mg/kg as recommended by clinical guidelines; This approach therefore reflects actual prescribing and documentation practices in our setting rather than an attempt to replace weight-based pharmacological principles.*

*Limitation: Body weight data were largely unavailable, preventing normalization of dose on a mg/kg basis and potentially introducing misclassification bias when categorizing fixed-dose regimens.”*

***Comment 2:***

The fact that the "high dose" group had the highest rates of complications like ARDS, DIC, and MODS before matching strongly suggests they were the sickest cohort. The text states these were similar after matching, but the data is not shown in Table 2 (which is pre-match). it is recommended provide a table of baseline characteristics after matching for the main comparison (Low vs. Usual) to prove adequate balance was achieved. Discuss residual confounding as a key limitation.

***Response:***

We thank the reviewer for this constructive suggestion. We agree that presenting baseline characteristics both before and after matching is important to demonstrate covariate balance. In the revised manuscript, we have added an additional supplementary table II and also clinical outcomes table IIIb (post-matching), which shows post-matching characteristics for each comparison group. After matching, all standardized mean differences were <0.1, indicating adequate balance. We have also mentioned a line in the limitations section to highlight that, despite matching, residual confounding remains possible given the observational design and the influence of physician discretion on dosing decisions.

***Comment 3:***

The primary causes for dose adjustment is to minimize polymyxin toxicity. It is a major oversight not to report rates of AKI, neurotoxicity, or other adverse events between the groups. A lower dose that is equally effective, but less toxic is practice-changing; one that is equally effective but with unknown safety is merely an observation. it is recommended, the authors must analyze and report comparative toxicity data. This is a non-negotiable requirement for a paper on antibiotic dosing.

***Response:***

We thank the reviewer for highlighting this important point. We fully agree that the evaluation of toxicity is critical when comparing different polymyxin B dosing strategies, particularly since lower dosing is often advocated to reduce nephrotoxicity and neurotoxicity. We have now clarified this explicitly in the Methods, Limitations and future implications sections.

*“Methods: All available data on the safety and effectiveness parameters of polymyxin B were retrieved from patient’s medical records and systematically analyzed.*

*Discussion: We observed all cases of acute kidney injury (AKI) reported in the medical records following the initiation of polymyxin B therapy. However, due to the retrospective design of the study, it was not possible to determine whether the reported AKI was attributable to the disease itself or to the drug. Furthermore, a causal relationship between AKI and polymyxin B administration could not be established, as no dechallenge or rechallenge was performed. This limitation is particularly important given the critical condition of the study population, in which multiple concurrent factors could have contributed to renal dysfunction.*

*Limitations: Furthermore, pharmacokinetic data to correlate administered doses with serum drug concentrations or toxicity endpoints were lacking, and systematic assessment of nephrotoxicity and neurotoxicity could not be performed. The absence of these data precluded evaluation of exposure-response relationships and dose-related toxicity.*

*Future implications: Also, they could incorporate standardized toxicity endpoints to better delineate the benefit-risk profile of different dosing strategies.”*

***Comment 4:***

The microbiological clearance rate is surprisingly low across all groups (~12-15%). This raises questions about the definition used ("eradication... with no subsequent growth of a more resistant organism" is very strict) or the timing of follow-up cultures. The statement that microbiological clearance was "better with low dosing" in the dialysis subgroup seems to contradict Table 4 and the text in section 3.3, which state no significant difference. This must be clarified. please provide more detail on how microbiological clearance was assessed. Report the median time to follow-up culture. The discrepancy in the dialysis subgroup results must be resolved.

***Response:***

We thank the reviewer for these helpful observations. We agree that the low clearance rates likely reflect the severity of infections in our population. Patients without repeat cultures were conservatively classified as “not cleared,” which may have further lowered observed rates. To address this, we have revised the Methods to provide greater detail on how microbiological clearance was defined and to report the median time to follow-up culture. Also, the table for dialysis subgroup is presented in Supplementary table III, which shows a significant difference between the groups.

*“Methods: Microbiological clearance was defined as the absence of the baseline pathogen in follow-up cultures obtained ≥72 hours after starting polymyxin B therapy. with no subsequent isolation of the same organism* [2]*. Patients without repeat cultures were conservatively classified as “not cleared”. This stringent definition was chosen to minimize misclassification, but it may underestimate clearance compared with more permissive criteria.*

*Results: Clearance was very low across all dosing groups (12-15%), which likely reflects the severity of the infections and the inherent difficulty of achieving eradication in critically ill patients. In many cases, polymyxin B was initiated as salvage therapy at an advanced stage of sepsis, by which time microbiological clearance was rarely observed.”*

***Comment 5:***

The handling of missing data (<20% imputed with median) is reasonable for a retrospective study but should be explicitly stated as a limitation.

***Response:***

We thank the reviewer for this helpful suggestion. We have now explicitly acknowledged in the Limitations that, although the imputation approach, we used (<20% imputed with median/mode) is standard for retrospective studies, it may still introduce bias.

*“Limitation: Although, missing data were handled by excluding variables with >20% missingness and imputing medians (continuous variables) or modes (categorical variables) when <20% was missing, this approach may still introduce bias and should be acknowledged as a limitation.”*

***Comment 6:***

The discussion defends the use of fixed-dose categories as reflecting "real-world prescribing practices" and being "easier to implement." While this may be true, it is a weak justification for abandoning scientific and pharmacological principles.

***Response:***

We thank the reviewer for this important observation. We fully agree that guideline-based dosing of polymyxin B is weight-adjusted, and that fixed-dose categories do not adhere to pharmacological principles. In our dataset, however, body weight was not consistently documented, which made it impossible to re-analyse doses as mg/kg. We have therefore revised the text to clarify that our use of fixed-dose categories reflects documentation realities in our setting rather than a preferred scientific approach. We have also emphasized this as a limitation in the Discussion and highlighted the need for future studies with complete weight-based and pharmacokinetic data to properly address exposure–response relationships.

*“Methods: We used fixed-dose categories because patient-level body weight data were not consistently available in the medical records. Moreover, it was often impractical to obtain accurate weight measurements for patients admitted to the ICU in critical condition due to the unavailability of high end patient beds in the ICU, thereby precluding dose recalculation in mg/kg as recommended by clinical guidelines; This approach therefore reflects actual prescribing and documentation practices in our setting rather than an attempt to replace weight-based pharmacological principles.*

*Discussion: The use of fixed-dose categories, while reflective of local practice, deviates from guideline-recommended weight-adjusted dosing and underscores the need for future studies with complete dosing, body weight, and pharmacokinetic data to define true exposure-response relationships.”*

***Comment 7:***

The discussion argues for "individualized, lower dosing strategies" and "minimizing toxicity" but provides zero data to support the claim that their low-dose strategy is actually less toxic. This is a critical logical leap.

***Response:***

We thank the reviewer for this valuable observation. We agree that our study did not include systematic toxicity outcomes and that it would be inappropriate to claim a direct safety advantage for the lower-dose strategy. We have revised the Discussion and Conclusion to remove any suggestion of proven toxicity reduction and now explicitly state that our findings are hypothesis-generating.

*“Discussion: Furthermore, pharmacokinetic data to correlate administered doses with serum drug concentrations or toxicity endpoints were lacking, and systematic assessment of nephrotoxicity and neurotoxicity could not be performed. The absence of these data precluded evaluation of exposure-response relationships and dose-related toxicity.*

*Conclusion: In this single-centre retrospective study, lower fixed doses of polymyxin B were associated with decreased mortality and better clinical outcomes when compared with higher doses. They may be feasible in critically ill patients with renal impairment, with limited safety data. However, given the inherent limitations of observational research, including unmeasured confounding and absence of systematic toxicity assessment, these results should be interpreted as hypothesis-generating rather than practice-changing. Our findings support the feasibility and importance of investigating lower dose polymyxin B regimens in future RCTs.”*

***Comment 8:***

The claim that low dose "was associated with significantly improved 28-day survival" (line 379) is too strong. The more accurate conclusion from a retrospective study is "was associated with significantly lower mortality" or "was associated with significantly improved survival." The word "improved" can imply a causative intervention.

***Response:***

We thank the reviewer for highlighting this important point. We agree that our original wording could imply a causal effect, which is not appropriate in the context of an observational retrospective study. We have revised the text to use more neutral language (“associated with lower 28-day mortality”) and clarified in the Conclusion that our findings represent associations rather than proof of causality.

***Comment 9:***

Table 2 a pre-match table. A post-match table for the primary comparison is essential.

***Response:***

Thank you for pointing out regarding the post-matching tables on baseline and clinical outcomes variables. We have added this table as supplementary II and Table IIIb.

***Comment 10:***

Regarding table 3 and 4, Referencing is incorrect in the manuscript. Table 3 is pre-match outcomes, but the text (lines 271, 298) refers to a "Table 4" for post-match outcomes that doesn't exist in the provided text. The tables need to be renumbered and referenced correctly.

***Response:***

Thank you for pointing out the incorrect table referencing. We discovered that Table 3 (pre-match outcomes) was correctly included but the post-match outcomes table was not present in the submitted main text. The manuscript text has been updated so that the Results section now refers to Table 3a for pre-match outcomes and to Table 3b for post-match outcomes. All table captions and in-text references have been verified for consistency in the revised manuscript.

**Reviewer #3:**

***Comment 1:***

Retrospective nature: The authors acknowledge this, but more detail is needed about how missing or inconsistent clinical data were handled (e.g., infection source documentation, microbiological clearance definitions).

***Response:***

We thank the reviewer for this helpful suggestion. We have expanded the Methods to provide more detail on how missing and inconsistent data were addressed. Specifically, variables with >20% missingness were excluded, and those with <20% missingness were imputed (median for continuous, mode for categorical). Infection source documentation was adjudicated by two independent professionals, with cases lacking sufficient information classified as “undocumented.” For microbiological clearance, we have clarified that patients without follow-up cultures were conservatively classified as “not cleared.” We have also noted in the Limitations that these retrospective challenges may have introduced misclassification.

*“Methods: Clinical and microbiological data were extracted retrospectively from electronic medical records. When clinical documentation was inconsistent (eg. Infection source not specified or recorded differently across notes), we used the consensus of two independent reviewers (clinical pharmacist and critical care physician) to adjudicate the most likely source or classified it as “undocumented” if no consensus could be reached. Dialysis modality was recorded from patient charts and categorized as sustained low efficiency dialysis (SLED), continuous renal replacement therapy (CRRT) or intermittent hemodialysis (IHD).*

*Limitations: Microbiological data such as infection source, MIC values and timing of follow-up cultures were not uniformly documented, which may have introduced classification bias when assessing clearance.”*

***Comment 2:***

Exposure categorization: The rationale for defining "low," "usual," and "high" dosing cut-offs (Table I) should be better justified with reference to guideline standards or prior clinical literature. At present, these thresholds appear somewhat arbitrary.

***Response:***

We thank the reviewer for this constructive comment. We have now expanded the Methods to better justify the rationale for our exposure categories. The cut-offs used (<150 mg/15 LU, 150 mg/15 LU, and >150 mg/15 LU) were chosen because they reflected the most common fixed-dose regimens prescribed in our centre. We have clarified that this differs from guideline-recommended mg/kg dosing but is consistent with real-world practice in similar settings as reported in prior observational studies[1]. We have also acknowledged in the Limitations that this categorization may not align fully with guideline standards.

“*Methods: Dosing categories were defined according to the prescribing practices at our centre, where doses are routinely documented in lakh units (LU) rather than weight-based regimens. For categorization, we adopted thresholds reflecting the most frequently observed patterns in clinical practice. We categorized polymyxin B dosing into three strategies- “low”, “usual” and “high”- with specific loading and maintenance regimens as defined in Table I. Although these definitions differ from international guideline recommendations, [weight-based dosing (LD 2.0-2.5 mg/Kg; MD1.25-1.5mg/kg q12h)], they are consistent with prior observational studies that have reported fixed-dose regimens in similar settings*[1]*. Unfortunately, patient-level body weight was not consistently recorded in our dataset, preventing a formal mg/kg-based analysis. Patients were classified according to the predominant dosing regimen used during therapy. In cases where loading and maintenance doses differed (eg. High loading dose but usual maintenance dose), categorization was based on the maintenance regimen, as this reflects ongoing exposure. We used fixed-dose categories because patient-level body weight data were not consistently available in the medical records. Moreover, it was often impractical to obtain accurate weight measurements for patients admitted to the ICU in critical condition due to the unavailability of high end patient beds in the ICU, thereby precluding dose recalculation in mg/kg as recommended by clinical guidelines; This approach therefore reflects actual prescribing and documentation practices in our setting rather than an attempt to replace weight-based pharmacological principles.*

*Limitation: Body weight data were largely unavailable, preventing normalization of dose on a mg/kg basis and potentially introducing misclassification bias when categorizing fixed-dose regimens.”*

***Comment 3:***

Dialysis subgroup: Although subgroup analyses are presented, the heterogeneity of dialysis modalities (SLED, CRRT, HD) is high. The manuscript should provide more clarity on how these modalities were distributed across groups and whether these confounded outcomes.

***Response:***

We thank the reviewer for highlighting this important point. We have now clarified in the Methods how dialysis modalities were classified. While no major imbalance was observed, we agree that heterogeneity in dialysis practices may have influenced drug exposure and outcomes. We have acknowledged this as a limitation, since modality-specific clearance could not be fully adjusted for in our retrospective dataset.

*“Methods: Dialysis modality was recorded from patient charts and categorized as sustained low efficiency dialysis (SLED), continuous renal replacement therapy (CRRT) or intermittent hemodialysis (IHD).*

*Results: Among patients requiring dialysis, modalities included SLED (n= 93), CRRT (n= 9), both HD and SLED (n= 141) and HD (n= 29). Their distribution across dosing groups is shown in Table II. No significant imbalance in dialysis modality was observed between groups. Also, no significant differences were observed between groups for any baseline covariate after matching (p>0.05), indicating adequate balance (supplementary table II).*

*Limitation: The dialysis subgroup was heterogenous, comprising patients receiving SLED, CRRT and IHD, which prevented in performing further analysis after matching; differences in drug clearance between modalities may have contributed to residual confounding that could not be fully accounted for in our analysis.”*

***Comment 4:***

Confounders: Propensity score matching was performed, but key covariates (e.g., pathogen MIC values, concurrent nephrotoxic drugs) were not included. The authors should discuss how unmeasured confounding might influence results.

***Response:***

We appreciate this insightful comment. We agree that several important confounders, such as pathogen MIC values and exposure to concurrent nephrotoxic agents, were not consistently available in our dataset and therefore could not be included in the propensity score model. MIC values were only performed by the clinical microbiology upon request by the treating physicians and not directly available for all patients, which is a practice of diagnostic stewardship. We have now explicitly discussed this in the Limitations section, acknowledging that these unmeasured factors could have influenced both dose selection and outcomes. We have clarified that, despite adjustment for key measured covariates, residual confounding remains a significant concern, and our findings should be interpreted as hypothesis-generating.

*“Discussion: Additionally, microbiological parameters such as MIC values for polymyxin B and data on concurrent nephrotoxic agents (eg., aminoglycosides, vancomycin, loop diuretics) were not consistently available and could not be incorporated into the model. Both factors could plausibly influence treatment selection and patient outcomes; therefore, the observed associations should be interpreted as hypothesis-generating rather than casual.”*

***Comment 5:***

The manuscript uses Cox regression, Kaplan–Meier survival, and logistic regression appropriately. However:

o Confidence intervals should be reported consistently for all effect sizes (not only HRs).

o The choice of caliper width for propensity score matching (0.1 SD) should be justified.

o Post-hoc power analysis adds little value; more emphasis should be placed on observed effect sizes and their precision.

***Response:***

We thank the reviewer for these valuable statistical suggestions. We have now ensured that all effect sizes (hazard ratios, odds ratios) are reported with their corresponding 95% confidence intervals throughout the text and tables for consistency. The Methods section has been updated to justify our use of a 0.1 SD caliper width for propensity score matching. Finally, we have minimized discussion of post-hoc power analysis and have instead emphasized the interpretation of observed effect sizes and their confidence intervals as measures of clinical and statistical precision.

*“Methods: The propensity score, reflecting each patient’s probability of assignment to a given dosing strategy (LD, UD or HD), facilitated pairwise 1:1 nearest-neighbour matching without replacement (“LD vs UD”, “UD vs HD”, LD vs HD”) with a caliper of 0.1 SD of the logit of the score. This caliper was chosen based on established recommendations from simulation studies by Austin et al.,*[3] *which suggest that a 0.1 SD caliper achieve optimal balance while minimizing bias and variance in most clinical datasets.*

*Discussion: Also, rather than focusing on post-hoc power, we emphasize the observed effect sizes and their corresponding 95% confidence interval as measures of both statistical precision and clinical relevance.”*

***Comment 6:***

The finding that usual dosing was associated with higher mortality than low dosing is counterintuitive. The authors should explore potential explanations (e.g., confounding by severity, higher toxicity, clinician bias in assigning higher doses to sicker patients).

***Response:***

We thank the reviewer for this thoughtful observation. We agree that the higher mortality observed in the usual-dose group is counterintuitive and warrants further explanation. We have now expanded the Discussion to explore several plausible contributing factors, including confounding by indication (clinicians selecting higher doses for critical patients), potential dose-related toxicity in those with renal impairment, and dosing variability due to fixed-dose prescribing practices. We have clarified that these mechanisms are speculative and highlight the need for prospective, controlled studies to verify causality.

*“Discussion: The observation that patients receiving the usual-dose regimen experienced higher mortality compared with those on lower doses was unexpected. This finding likely reflects a combination of clinical and pharmacological factors rather than a true dose-response effect. Confounding by indication is the most plausible explanation, as clinicians may have preferentially prescribed higher doses to patients perceived as more severely ill or with poor prognostic indicators not fully captured by APACHE II or SOFA scores. Additionally, the potential for dose-related adverse effects, particularly nephrotoxicity in patients with renal impairment, cannot be excluded. Local fixed dose prescribing practices may also have resulted in inadvertent overdosing in smaller patients, amplifying toxicity risk. Collectively, these factors could explain the paradoxical association and underscore the need for randomized controlled trials to delineate true dose-outcome relationships.”*

***Comment 7:***

Microbiological clearance data are limited; since clearance rates were low across groups, the conclusion of "non-inferiority" seems overstated. The authors should reframe this as "no significant difference observed" rather than formal non-inferiority.

***Response:***

We thank the reviewer for this important clarification. We agree that due to limited microbiological clearance data, and low clearance rates across groups, the findings do not support a formal claim of non-inferiority. We have therefore revised the Results, Discussion, and Conclusion sections to use more appropriate terminology such as “no significant difference observed” and “comparable outcomes”. These changes ensure that our conclusions accurately reflect the exploratory, hypothesis-generating nature of the study.

***Comment 8:***

The discussion is generally balanced but occasionally overstates the strength of evidence. Phrases like “supports individualized, lower dosing strategies” should be qualified as hypothesis-generating pending prospective confirmation. Comparison with other recent clinical and pharmacokinetic studies (e.g., therapeutic drug monitoring-guided trials, 2022–2024) could be expanded.

***Response:***

We thank the reviewer for this excellent suggestion. We have revised the Discussion and Conclusion to adopt a more cautious tone, clarifying that our findings are hypothesis-generating and not confirmatory. Statements implying definitive clinical recommendations have been rephrased to emphasize the need for prospective validation. In addition, we have expanded the Discussion to incorporate recent (2022–2024) literature on therapeutic drug monitoring and pharmacokinetic-guided polymyxin B dosing, providing context for our findings within the evolving evidence base.

*“Discussion: Recent pharmacokinetic and TDM-focused studies emphasize the large interpatient variability in polymyxin B exposure and the potential utility of TDM or PK-guided dosing to optimise therapeutic windows. Clinical TDM studies and population-PK analyses have shown that model-based dose adjustment improves target attainment and highlight the limitations of fixed-dose approaches, particularly in special populations such as those receiving extracorporeal support or renal replacement therapy* [4, 5]*.”*

***Comment 9:***

Abstract: The results section should report actual mortality percentages in each group for clarity.

Figures/Tables: Figures lack detailed legends, making them difficult to interpret independently.

Terminology: Ensure consistency in abbreviations (e.g., “LU” vs. “lakh units”; sometimes spelled inconsistently).

References: The reference list is up to date, but some statements in the Discussion (e.g., inter-individual variability in Polymyxin B pharmacokinetics) could benefit from citing very recent studies (2023–2025).

***Response:***

We thank the reviewer for these constructive editorial suggestions.

• The Abstract has been revised to include the actual 28-day mortality percentages for each dosing group, improving clarity and transparency.

• All figure and table legends have been expanded to provide full descriptions of variables, statistical tests, and abbreviations, ensuring each is interpretable independently.

• Terminology has been standardized across the manuscript by defining “LU (lakh units)” at first mention and using the abbreviation “LU” thereafter.

• Finally, we have incorporated recent 2023–2025 pharmacokinetic and TDM-guided studies (Liu et al., 2023; Liang et al., 2023; Zeng et al., 2024; Zheng et al., 2023) into the Discussion to strengthen and update the context regarding inter-individual variability and PK-guided dosing strategies.

**Reviewer #4:**

***Comment 1:***

Line 159: Please add the reference for this definition: “Multidrug resistance was defined as the non-susceptibility to at least one antibiotic agent in ≥3 antimicrobial classes.”

***Response:***

We thank the reviewer for this helpful observation. We have now added the appropriate reference to support this definition.

*“Multidrug resistance was defined as the non-susceptibility to at least one antibiotic agent in ≥3 antimicrobial classes*[6]*.”*

***Comment 2:***

Discussion:

1.You only mentioned one previous study (Lie et al.) regarding 28-day mortality between high and low-dose polymyxin B groups. Are there no other previous studies related to that? If there are other studies, please add them.

2. Are there also no other previous studies related to other findings? If there are studies, please add them.

***Response:***

We thank the reviewer for this thoughtful comment. We have now expanded the discussion to include relevant studies supporting our observations.

*“Discussion: Several additional studies similarly explore mortality outcomes in relation to polymyxin B. Rigatto et al.,*[7] *reported that daily doses ≥150mg/day were associated with increased AKI but not definitively with higher mortality. In patients with bloodstream infections due to carbapenem-resistant gram-negative rods, doses <1.3 mg/kg/day were associated with greater 30-day mortality compared to higher doses*[8]*. Additionally, a high-dose cohort study using ~30,000 IU/kg/day polymyxin B found promising survival but at the cost of significant AKI incidence*[9]*. In contrast, our findings indicate that low-dose regimens were non-inferior to the usual dose in terms of 28-day mortality, given the variations in patient selection, severity of illness, or local resistance patterns. Cai et al., reported microbiological eradication in 95.8% of bloodstream infections treated with high dose polymyxin B*[8]*. Microbiological clearance rates in our study did not significantly differ across groups, suggesting that lower doses may be sufficient to achieve bacteriological efficacy in selected populations, especially when combination therapy is employed.”*

***Comment 3:***

***Minor comments:***

1. Abstract: (HR:1.47(1.11-1.95);p=0.007) is better to be written as (HR=1.47; 95%CI = [1.11-1.95];p=0.007).

2. The dot (.) must be written after the reference number, like this [ ].

***Response:***

We thank the reviewer for pointing out these important formatting inconsistencies. We have corrected the statistical reporting style in the Abstract and throughout the manuscript to read “HR = 1.47; 95% CI [1.11–1.95]; p = 0.007” as per PLOS ONE formatting standards. Reference punctuation has also been standardized so that the period follows the reference number (e.g., “…as previously described [12].”). These edits have been applied consistently across the manuscript and supplementary materials.

**References:**

1. Liu S, Wu Y, Qi S, Shao H, Feng M, Xing L, et al. Polymyxin B therapy based on therapeutic drug monitoring in carbapenem-resistant organisms sepsis: the PMB-CROS randomized clinical trial. Crit Care. 2023;27:232. https://doi.org/10.1186/s13054-023-04522-6.

2. Nazer L, Baseem Al Rihani S, Hawari F, Le J. Microbiological clearance and nephrotoxicity associated with high-dose colistimethate sodium. 2013. https://doi.org/10.1097/01.ccm.0000440145.17469.a7.

3. Austin PC. The use of propensity score methods with survival or time-to-event outcomes: reporting measures of effect similar to those used in randomized experiments. Stat Med. 2014;33:1242–58. https://doi.org/10.1002/sim.5984.

4. Liang D, Liang Z, Deng G, Cen A, Luo D, Zhang C, et al. Population pharmacokinetic analysis and dosing optimization of polymyxin B in critically ill patients. Front Pharmacol. 2023;14:1122310. https://doi.org/10.3389/fphar.2023.1122310.

5. Zheng Y, Xu B, Chen S, Liu M, Huang H, Wang J, et al. Population Pharmacokinetic Modeling Using Polymyxin B Free Plasma Concentrations From Published Reports and Evaluation of Dosage Regimens Based on Monte Carlo Simulation in Critically Ill Patients. J Clin Pharmacol. 2023;63:1036–44. https://doi.org/10.1002/jcph.2261.

6. Magiorakos A-P, Srinivasan A, Carey RB, Carmeli Y, Falagas ME, Giske CG, et al. Multidrug-resistant, extensively drug-resistant and pandrug-resistant bacteria: an international expert proposal for interim standard definitions for acquired resistance. Clin Microbiol Infect. 2012;18:268–81. https://doi.org/10.1111/j.1469-0691.2011.03570.x.

7. Rigatto MH, Behle TF, Falci DR, Freitas T, Lopes NT, Nunes M, et al. Risk factors for acute kidney injury (AKI) in patients treated with polymyxin B and influence of AKI on mortality: a multicentre prospective cohort study. J Antimicrob Chemother. 2015;70:1552–7. https://doi.org/10.1093/jac/dku561.

8. Cai Y, Leck H, Tan RW, Teo JQ, Lim T-P, Lee W, et al. Clinical Experience with High-Dose Polymyxin B against Carbapenem-Resistant Gram-Negative Bacterial Infections-A Cohort Study. Antibiotics (Basel). 2020;9:451. https://doi.org/10.3390/antibiotics9080451.

9. Nelson BC, Eiras DP, Gomez-Simmonds A, Loo AS, Satlin MJ, Jenkins SG, et al. Clinical outcomes associated with polymyxin B dose in patients with bloodstream infections due to carbapenem-resistant Gram-negative rods. Antimicrob Agents Chemother. 2015;59:7000–6. https://doi.org/10.1128/AAC.00844-15.
