# Supplementary material for: Clinical outcomes with lower versus conventional dose polymyxin B regimens in dialysis dependent and non-dialysis patients with gram-negative sepsis: A real-world propensity-score matched cohort study
Source: PLoS One. 2026 Mar 4;21(3):e0342835. doi: 10.1371/journal.pone.0342835 (PMC12959684; doi:10.1371/journal.pone.0342835)
Supplement: Attachment — Submitted filename: Response to reviewers-2.docx [file pone.0342835.s014.docx]

**Point-by-point response to reviewer comments**

**Reviewer #1:**

**Comment 1:**

Address the Fixed-Dosing Limitation: This is the top priority. The manuscript must explicitly state, in both the methods and limitations sections, that the lack of weight-based dosing is a major constraint on the interpretability and generalizability of the results. The categories are center-specific constructs.

***Response:***

We thank the reviewer for highlighting this critical issue. We have now explicitly acknowledged the absence of weight-based dosing as a major methodological limitation affecting both interpretability and generalizability. In the methods section, we clarify that polymyxin B dosing categories were derived from centre-specific fixed-dose prescribing practices, driven by routine documentation in lakh units and the unavailability of reliable body weight data in critically ill ICU patients. We emphasize that these categories represent real-world prescribing constructs rather than pharmacologically validated, weight-based regimens.

In the limitations section, we further highlight that the inability to normalize doses on a mg/kg basis precludes direct comparison with guideline-recommended dosing strategies and may introduce exposure misclassification. This constraint is now clearly stated as a major limitation of the study and reinforces that our findings should be interpreted as hypothesis-generating rather than practice-changing.

*Methods: Polymyxin B dosing in our centre is routinely prescribed and documented in fixed lakh-unit doses rather than as weight-based regimens. Accurate body weight was not consistently available for critically ill ICU patients due to clinical instability and logistical constraints, precluding recalculation of doses on a mg/kg basis. Consequently, dosing categories (‘low’, ‘usual’ and ‘high’) were derived from centre-specific prescribing patterns and reflect real-world practice* (1) *rather than guideline-recommended, weight-adjusted dosing strategies. This approach captures actual clinical decision-making in our setting but limits direct pharmacokinetic interpretation and comparison with international dosing recommendations.*

*Limitations: An important limitation of this study is the absence of body weight for a substantial proportion of patients, which prevented normalization of polymyxin B dosing on a mg/kg basis. As a result, the fixed-dose categories used in this analysis represent centre-specific prescribing constructs rather than pharmacologically standardized dosing thresholds. This limitation restricts the interpretability of dose-response relationships and limits the generalizability of our findings to settings where weight-based dosing is routinely implemented. Accordingly, the observed associations should be interpreted cautiously and viewed as hypothesis-generating.*

**Comment 2:**

Incorporate Safety Data: The study is incomplete without an analysis of nephrotoxicity. The authors must go back to the data, apply a standard AKI definition (e.g., KDIGO), and report the incidence of AKI across the dosing groups. If the data is truly unavailable, this must be stated as a critical limitation that precludes any conclusions about the safety of lower doses.

***Response:***

We fully agree with the reviewer that nephrotoxicity is a key safety outcome when evaluating polymyxin B dosing strategies. We carefully re-evaluated the available dataset to determine whether a standardized AKI definition could be applied. However, KDIGO-based AKI classification could not be reliably performed because serial serum creatinine values and urine output measurements were inconsistently documented at the required time points, and many patients initiated renal replacement therapy early during their ICU course.

Consequently, while AKI events were recorded as part of routine clinical documentation, these could not be adjudicated using standardized criteria nor casually attributed to polymyxin B exposure versus sepsis-related organ dysfunction or hemodynamic instability. We have therefore explicitly stated this as a critical limitation in the methods and limitations sections and clarified that the absence of standardized nephrotoxicity assessment precludes any conclusions regarding the safety or toxicity advantages of lower-dose regimens. The manuscript has been revised accordingly to avoid any safety-related inference.

*Methods: Although AKI events were extracted from clinical records, standardized adjudication using KDIGO criteria was not feasible because serial serum creatinine measurements and urine output data were inconsistently available at required time points, particularly in patients with early initiation of renal replacement therapy.*

*Results: These AKI events reflect clinician documented diagnoses rather than standardized KDIGO-defined nephrotoxicity and should therefore be interpreted descriptively rather than as a formal safety comparison across dosing groups.*

*Limitations: AKI events, though documented in medical records, could not be reliably attributed to polymyxin B exposure versus sepsis-related organ dysfunction or hemodynamic instability. This precludes any conclusions regarding the comparative safety or nephrotoxicity advantage of lower versus higher polymyxin B dosing regimens and our findings should not be interpreted as evidence of reduced toxicity.*

**Comment 3:**

Improve Statistical Reporting:

i. Remove the post-hoc power calculation.

***Response:***

We thank the reviewers for this suggestion. All post-hoc power calculations have been completely removed from the manuscript. Consistent with best statistical practice, we now focus on reporting effect estimates with corresponding 95% confidence intervals to convey the magnitude and precision of the observed associations.

ii. Provide a table of post-matching covariate balances with Standardized Mean Differences (SMDs) to prove the effectiveness of the PSM.

***Response:***

We thank the reviewer for this important suggestion. To formally demonstrate the effectiveness of the propensity score matching, we have now added a dedicated table reporting standardized mean differences (SMDs) for all covariates included in the propensity score model after matching. This table has been included as supplementary table II. Following matching, all covariates achieved acceptable balance with SMDs below 0.1, indicating adequate covariate balance across dosing groups.

iii. Ensure all p-values and confidence intervals are reported consistently.

***Response:***

We thank the reviewer for this comment. We have carefully reviewed the entire manuscript and standardized the reporting of all p-values and confidence intervals. P-values are now reported consistently using lowercase “p” and effect estimates (hazard ratios and odds ratios) are presented with corresponding 95% confidence intervals in a uniform format throughout the text, tables and figures. These revisions have been applied across the abstract, results, tables and supplementary materials.

**Comment 4:**

Refine the Discussion and Conclusions:

i. Tone down definitive language. Use "associated with" instead of "caused" or "led to."

***Response:***

We thank the reviewer for this important interpretative suggestion. We have revised the discussion and conclusions to remove definitive or causal language throughout the manuscript. All statements implying causation (eg., “caused”, “led to”, “resulted in”) have been replaced with associative terminology (eg., “was associated with”, “was observed with”, “correlated with”), consistent with the observational nature of the study. The conclusions have been correspondingly softened to emphasize associations rather than causality.

ii. Remove any claim of "non-inferiority" or "fewer adverse events" unless supported by data.

***Response:***

We agree with the reviewer and have removed all claims implying non-inferiority or reduced adverse events. The manuscript has been carefully revised to eliminate terms such as “non-inferior”, “safer” and “fewer adverse events”, as formal non-inferiority testing and standardized toxicity assessments were not performed. All statements have been reframed to describe observed associations only, without implying equivalence or safety advantages.

iii. Frame the primary finding (higher mortality with usual dose) within the context of "confounding by indication" as the most plausible explanation.

***Response:***

We thank the reviewer for this important interpretative point. We have now explicitly framed the observed association between usual dose polymyxin B and higher mortality within the context of confounding by indication. In the discussion, we clarify that clinicians may have preferentially prescribed usual or higher doses to patients perceived as more severely ill or clinically deteriorating, based on factors not fully captured by APACHE II or SOFA scores. We emphasize that this residual confounding is the most plausible explanation for the observed association and that the findings should be interpreted as hypothesis-generating rather than indicative of a true causal or dose-dependent effect.

*Discussion: The finding of higher 28-day mortality among patients receiving the usual-dose polymyxin B regimen should be interpreted with caution. Confounding by indication is the most plausible explanation for this association. In routine clinical practice, clinicians may escalate polymyxin B dosing in patients perceived to have more severe illness, poor early response, or unfavourable prognostic features that are not fully captured by severity scores such as APACHE II or SOFA. Although propensity score matching was used to balance measured confounders, residual confounding from unmeasured clinical factors likely remains. Therefore, the observed association should not be interpreted as evidence of a harmful effect of usual-dose therapy but rather as a reflection of treatment selection bias inherent to retrospective observational studies.*

**Comment 5:**

Thorough Language and Copy-Editing: A native English speaker or professional editing service should review the entire manuscript to correct grammatical errors, improve sentence flow, and ensure consistent terminology.

***Response:***

We thank the reviewers for this suggestion. The manuscript has undergone comprehensive language editing to improve grammatical accuracy, sentence structure, clarity and consistency of terminology throughout the text, tables, figures and supplementary materials. Revisions were applied across all sections to enhance readability and ensure alignment with journal standards.

Final Verdict: The manuscript reports a valuable and interesting real-world analysis. With the major revisions outlined above particularly a more critical acknowledgment of its limitations and the inclusion of safety data—it has the potential to be a significant contribution to the literature. In its current form, it is not ready for publication but is a strong candidate for major revision.

***Response:***

We sincerely thank the reviewer for their thoughtful evaluation of our work and for recognizing the potential contribution of this real-world analysis. We have carefully addressed all major concerns raised, including a more critical and explicit acknowledgment of the study’s limitations, removal of unsupported interpretative claims, refinement of causal language, and transparent clarification regarding the unavailability of standardized safety (nephrotoxicity) assessment. We believe that the extensive revisions made in response to the reviewer’s detailed comments have substantially strengthened the methodological rigor, interpretative clarity, and overall quality of the manuscript.

**Reviewer #3:**

Overall, the authors have addressed my major methodological and interpretative concerns and substantially improved the manuscript. The handling of missing data, exposure categorization, dialysis subgroups, and unmeasured confounding is now described more transparently, and the statistical methods (including reporting of confidence intervals and justification of the propensity-score caliper) are clearer and more appropriate. The Discussion has been reframed with a more cautious, hypothesis-generating tone, with better integration of recent PK/TDM literature, and the limitations around MIC data, toxicity assessment, and single-centre generalizability are now explicitly acknowledged. The abstract now reports group-specific mortality, tables/figures are clearer, terminology is more consistent, and the reference on MDR definitions has been added. I would recommend acceptance for this manuscript.

***Response:***

We sincerely thank the reviewer for their careful reassessment of the manuscript and for the positive and constructive feedback. We are pleased that the revisions have addressed the methodological and interpretative concerns, and that the improvements in transparency, statistical reporting, discussion framing, and acknowledgment of limitations were found to be appropriate. We appreciate the reviewer’s time and consideration and have retained these revisions in the current version of the manuscript.

**References**

1.	Liu S, Wu Y, Qi S, Shao H, Feng M, Xing L, et al. Polymyxin B therapy based on therapeutic drug monitoring in carbapenem-resistant organisms sepsis: the PMB-CROS randomized clinical trial. Crit Care. 2023 June 13;27(1):232.
